# Imbalance of gut microbiota in gestational diabetes

**DOI:** 10.1186/s12884-024-06423-0

**Published:** 2024-04-01

**Authors:** Weiyi Shen, Qianyi Chen, Renbin Lin, Zhefang Hu, Man Luo, Yanwei Ren, Keren Huang, Li Wang, Shujie Chen, Lan Wang, Yu Ruan, Lijun Feng

**Affiliations:** 1 Department of Pathology, Sir Run Run Shaw Hospital, Zhejiang University School of Medicine, Hangzhou, 310016 Zhejiang Province China; 2 Department of Gastroenterology, Sir Run Run Shaw Hospital, Zhejiang University School of Medicine, Hangzhou, 310016 Zhejiang Province China; 3 Prevention and Treatment Research Center of Senescent Disease, Zhejiang University School of Medicine, Hangzhou, 310058 Zhejiang Province China; 4 Department of Nutriology, Sir Run Run Shaw Hospital, Zhejiang University School of Medicine, Hangzhou, 310016 Zhejiang Province China; 5 Department of Gastroenterology, Hangzhou Hospital of Traditional Chinese Medicine, Zhejiang Chinese Medicine University, Hangzhou, 310005 Zhejiang Province China; 6 Department of Obstetrics and Gynecology, Sir Run Run Shaw Hospital, Zhejiang University School of Medicine, Hangzhou, 310016 Zhejiang Province China; 7 Department of Endocrinology and Metabolism, Sir Run Run Shaw Hospital, Zhejiang University School of Medicine, Hangzhou, 310016 Zhejiang Province China

**Keywords:** Gestational diabetes, Gut microbiota, Protective compensation, Consequences of- damage, Faecalibacterium prausnitzii

## Abstract

**Aim:**

To investigate the differences in gut microbiota composition among nonpregnant women of reproductive age, healthy pregnant women, and gestational diabetes (GD) patients.

**Methods:**

A total of 45 outpatients were enrolled and divided into three groups: nonpregnant women of reproductive age (control group, *n* = 23), healthy pregnant women (normal group, *n* = 10), and GD patients (GD group, *n* = 12). Faecal samples were collected and sequenced using 16S rRNA gene sequencing to analyse the microbial composition.

**Results:**

(1) Pregnant patients exhibited an increase in the abundance of *Streptococcus* (P_normal_ = 0.01286, P_GD_ = 0.002965) and *Blautia* (P_normal_ = 0.0003924, P_GD_ = 0.000246) but a decrease in the abundance of *Roseburia* (P_normal_ = 0.0361, P_GD_ = 0.007075), *Phascolarctobacterium* (P_normal_ = 0.0003906, P_GD_ = 0.02499) and *Lachnoclostridium* (P_normal_ = 0.0003906, P_GD_ = 0.03866). (2) Compared with healthy pregnant women, GD patients had an excessive increase in *Streptococcus* abundance and decrease in *Roseburia* abundance. The increase in *Blautia* abundance and the decrease in *Phascolarctobacterium* and *Lachnoclostridium* abundance in GD patients were less than those in healthy pregnant women. (3) The abundance of *Faecalibacterium prausnitzii* decreased significantly in GD patients (P_GD_ = 0.02985) but not in healthy pregnant patients (P_normal_ = 0.1643).

**Conclusions:**

Abnormal increases and decreases in the abundances of gut microbiota components, especially *Faecalibacterium prausnitzii*, were observed in GD patients.

**Trial registration:**

The cross-sectional research was conducted in accordance with the Declaration of Helsinki, and approved by Sir Run Run Shaw Hospital Clinical Trials and Biomedical Ethics Committee. The study has been registered in the Chinese Clinical Trial Registry (ChiCTR1900026164, 24/09/2019, http://www.chictr.org.cn/showproj.aspx?proj=43,455).

**Supplementary Information:**

The online version contains supplementary material available at 10.1186/s12884-024-06423-0.

## Introduction

Gestational diabetes (GD) is a metabolic disorder that occurs during pregnancy. It causes hyperglycaemia during pregnancy but mostly resolves after birth. The incidence of GD is the highest among all complications of pregnancy. Individuals with GD can incur a high risk of excessive foetal growth, obesity, and cardiovascular disease [1]. Hence, exploring the pathogenesis and effective treatments of GD is clinically important.

Previous studies have highlighted the considerable potential of the gut microbiota in the regulation of metabolic balance and identified certain differences in the gut microbiota composition between GD patients and healthy pregnant women [2, 3]. Differences in specific gut microbiota compositions during the first trimester trigger insulin resistance and might be relevant to subsequent blood glucose disorders [4]. Previous studies have shown a direct correlation between intestinal bacteria and blood glucose levels. For example, *Bacteroides* exhibited a positive correlation with glucose levels, whereas *bifidobacteria* demonstrated a negative correlation [5, 6]. Clinical trials revealed similar results; modulating the microbiota by administering probiotics decreased fasting blood glucose levels [7]. Interestingly, the meconium and first faeces of newborns from mothers with GD who received insulin treatment exhibited alterations in microbiota, manifesting as a higher *Firmicutes/Bacteroidetes* (F/B) ratio [8]. While various changes in the relative abundance of the gut microbiota in women with GD have been investigated, few of these changes have been indicated as biomarkers. Moreover, no study has assessed the differences in the gut microbiota between nonpregnant women of reproductive age and pregnant women of two pregnancy statuses: normal pregnancy and GD pregnancy.

Environmental, genetic and pregnancy-related factors play vital roles in the pathogenesis of GD [9]. Pregnancy is accompanied by a high load of islets in the pancreas [10]. The placenta produces insulin enzymes, hormones (progestagens, oestrogens, and androgens), and antagonists of insulin, resulting in a shortage of insulin [11]. During pregnancy, the abundance of the gut microbiota, which adapts to maternal and foetal pregnancy demands, is altered [12]. However, whether these differences in abundance are protective (which are destroyed in GD patients, manifesting as deficient reductions in harmful bacteria or deficient increases in beneficial microbes in this research) or consequences of damage (which are magnified in GD patients, manifesting as excessive reductions in beneficial microbes or excessive increases in harmful bacteria in this research) have yet to be elucidated. The comparison of different alterations in the gut microbiota between healthy pregnant women and GD patients facilitates the further exploration of the cause of this condition.

In the present study, we compared the differences in the gut microbiota composition among nonpregnant women of reproductive age, healthy pregnant women, and GD patients and analysed the correlations between specific microbiota and blood glucose levels.

## Materials and methods

### Subjects

This was a single-centre, interventional study. A total of 23 nonpregnant women of reproductive age, 10 healthy pregnant women, and 39 GD patients (according to the International Diabetes Study Group (IADPSG) criteria [13]) were recruited from the outpatient clinic of Sir Run Run Shaw Hospital (Hangzhou, China) from July 2019 to December 2019. All subjects were required to provide their previous medical history and stool on the day of recruitment. In the GD group, 26 patients failed to provide stool in time, and 1 patient provided an insufficient sample. Ultimately, only 12 of these patients (GD patients) participated in subsequent intestinal bacteria detection and analysis. The trial has been registered in the Chinese Clinical Trial Registry (ChiCTR1900026164, 24/09/2019, http://www.chictr.org.cn/showproj.aspx?proj=43455).

### Inclusion criteria

#### Nonpregnant women of reproductive age

According to the diabetes diagnostic criteria, the inclusion criterion was age 18–49 years (inclusive) without a diagnosis of diabetes. The patients were willing to undergo intestinal bacteria detection and signed an informed consent form prior to participation in the study.

#### Normal pregnant women

According to the diagnostic criteria for GD [75 g oral glucose tolerance test (OGTT): fasting ≥ 5.1 mmol/L, 1 h ≥ 10 mmol/L, 2 h ≥ 8.5 mmol/L], the inclusion criteria were women aged 18–49 years (inclusive) without diagnosed GD with a gestational age ≥ 24 weeks and ≤ 32 weeks. The patients were willing to undergo intestinal bacteria detection and were included in the study after providing informed consent.

#### GD pregnant women

According to the GD diagnostic criteria (75 g OGTT: fasting ≥ 5.1 mmol/L, 1 h ≥ 10 mmol/L, 2 h ≥ 8.5 mmol/L), the inclusion criteria were women aged 18–49 years (inclusive) with a GD diagnosis and a gestational age of 24–32 weeks (inclusive). The patients were willing to undergo intestinal bacteria detection and were included in the study after providing informed consent.

### Exclusion criteria

The exclusion criteria were as follows: 1. diabetes (with symptoms of diabetes: fasting ≥ 7.0 mmol/L or random blood glucose ≥ 11.1 mmol/L or 75 g OGTT 2 h ≥ 11.1 mmol/L; without symptoms of diabetes: in addition to the above diagnostic criteria, also ① OGTT 1 h ≥ 11.1 mmol/L, ② second test fasting ≥ 7.8 mmol/L, ③ second test OGTT 2 h ≥ 11.1 mmol/L) or impaired fasting glucose (6.1 mmol/L ≤ fasting < 7.0 mmol/L) or impaired glucose tolerance (7.8 mmol/L ≤ OGTT 2 h < 11.1 mmol/L) before pregnancy; 2. polycystic ovary syndrome before pregnancy; 3. severely stressful life events, severe anxiety, and depression; 4. hypertension, chronic hypertension, chronic nephritis, autoimmune diseases or a history of other diseases; 5. chronic diarrhoea, a history of chronic gastrointestinal diseases, or functional gastrointestinal disease; and 6. the use of antibiotics or probiotics within a month.

### Clinical data and stool sample collection

Fasting blood glucose levels, 1-h OGTT results, 2-h OGTT results, and albumin, TG, TC, HDL, LDL, VLDL, inflammation marker and CRP levels were collected from the patients’ electronic medical records. Stool samples were obtained from outpatients at the Zhejiang University School of Medicine Sir Run Run Shaw Hospital on the day of recruitment. The patients collected their stool in sterile plastic tubes before leaving the hospital that same day, and then the samples were sent to the outpatient department immediately. The outpatient doctor placed them in a box with Drikold. We collected the samples within half an hour, and they were stored at − 80 °C until DNA extraction.

### 16S sequencing and bioinformatics analyses

DNA was extracted using the TIANamp Stool DNA Kit (Tiangen Biotech, Beijing, China). The DNA concentration and purity were detected on a Nanodrop 2000 UV‒vis spectrophotometer (Thermo Fisher Scientific, Waltham, America).

Metagenomic sequencing and bioinformatics analyses were performed by Majorbio BioPharm Technology (Shanghai, China). The V3-V4 variable regions of the 16S rRNA subunit gene were used in this study. The extracted genomic DNA was analysed by 1% agarose gel electrophoresis and amplified by polymerase chain reaction (PCR; ABI GeneAmp® 9700, America). Each sample was analysed in triplicate. The amplified products of the same sample were pooled and resolved by 2% agarose gel electrophoresis. Subsequently, the products were purified using an AxyPrep™ DNA Gel Extraction Kit (Axygen, Silicon Valley, America) and quantified on a Quantifluor™ ST Blue Fluorescence Quantitative System (Promega, Madison, America). A gene library was constructed using a TruSeq™ DNA Sample Prep Kit for Illumina. Finally, the amplified target DNA was sequenced by Majorbio on the Illumina MiSeq platform.

Paired-end reads obtained by MiSeq sequencing were spliced according to overlap correlation, the sequence quality was controlled, and the sequences were filtered. After the samples were distinguished, operational taxonomic unit (OTU) cluster analysis and taxonomic analysis were performed. The above statistical and visual analyses, such as multivariate analysis and significance tests, were performed for the microbial composition and phylogenetic information of multiple samples.

### Statistical analysis

The continuous data are presented as the mean ± standard error of the mean (SEM) and were analysed by unpaired t test, Welch’s t test or the Kolmogorov‒Smirnov test according to the normal distribution test (Shapiro‒Wilk normality test) and the homogeneity test for variance (F test). Categorical data are presented as percentages and were compared by the χ^2^ test. The consequences of bacterial detection were analysed on the Majorbio cloud platform. Operational taxonomic units (OTUs) were clustered with a 97% similarity cut-off using UPARSE (version 7.1; http://drive5.com/uparse/) based on the database silva 138 (primary database). The diversity of the communities was indicated by statistical indices (the ACEI for richness, Simpson’s index for diversity, and the Heip index for evenness). The differences in alpha (α)-diversity between GD patients and healthy control individuals were analysed by the Wilcoxon rank-sum test. The differences in β diversity between the two groups were determined by principal coordinates analysis (PCoA) and nonmetric multidimensional scaling (NMDS) analysis. The differences in the relative abundance of gut bacteria between GD patients and healthy control individuals at the genus level were calculated by the Wilcoxon rank-sum test. The differences in intestinal microbiota constituents ranging from the phylum to the genus level were analysed by linear discriminant analysis effect size (LEfSe).

## Results

### Characteristics of GD patients and healthy pregnant women

**Fig. 1:**
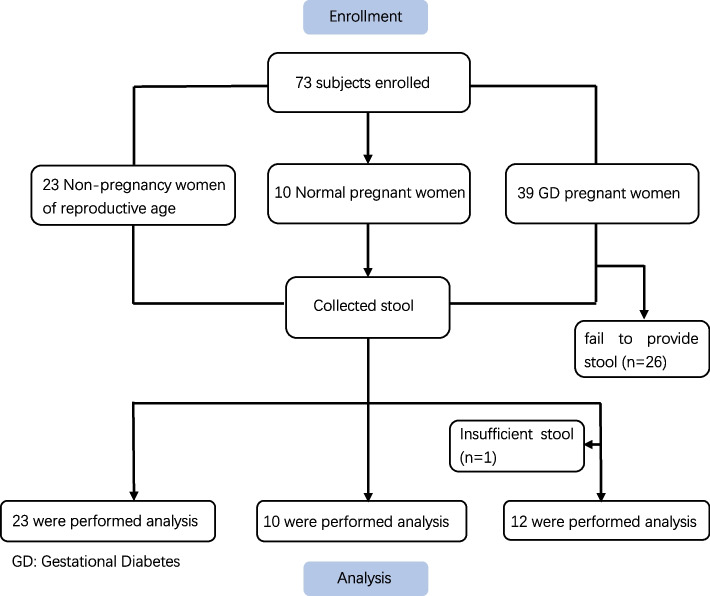
Study flowchart

A total of 23 nonpregnant women of reproductive age, 10 healthy pregnant women, and 12 GD patients were included in this study (Fig. 1).

The gestational BMI of GD patients was greater than that of individuals in the other groups (*p* = 0.005). No significant differences were observed in age, university degree or family history among the three groups (*p* > 0.05) (Table 1).

**Table 1:** Personal information of the GD patients, healthy pregnant women and nonpregnant women (BMI and age, mean ± SEM)

	GD patients	Normal pregnant women	Nonpregnant women	*P* value
Pregestational BMI	23.020 ± 2.557 (*n* = 12)	20.820 ± 1.501 (*n* = 10)	20.750 ± 2.559 (*n* = 23)	0.005
Age (years)	31.920 ± 4.833 (*n* = 12)	31.000 ± 2.944 (*n* = 10)	28.610 ± 4.008 (*n* = 23)	0.060
University degree	83.3% (10/12)	70% (7/10)	73.9% (17/23)	0.743
Family history				
Diabetes	33.3% (4/12)	10% (1/10)	17.4% (4/23)	0.358
Hypertension	16.7% (2/12)	30% (3/10)	17.4% (4/23)	0.668

*BMI* body mass index, *Age*, One-way ANOVA; *University degree/Family history*, Chi-square test, *GD *Gestational diabetes

Among the clinical parameters, triglyceride (TG) and C-reactive protein (CRP) levels were significantly greater in the GD patients than in the healthy pregnant women (TG: *p* = 0.042, CRP: *p* = 0.04) (Table 2). Regarding blood glucose, the main difference between the two groups was observed in postprandial blood glucose rather than fasting blood glucose (Table 2).

**Table 2:** Clinical characteristics of GD patients and healthy pregnant women (mean ± SEM)

	GD patients	Normal pregnant women	*P* value
Blood glucose			
Fasting blood glucose	4.960 ± 0.121 (*n* = 12)	4.724 ± 0.093 (*n* = 10)	0.1507
OGTT-1 h	10.120 ± 0.329 (*n* = 12)	7.070 ± 0.387 (*n* = 10)	< 0.0001
OGTT-2 h	8.643 ± 0.252 (*n* = 12)	6.391 ± 0.321 (*n* = 10)	< 0.0001
Nutriture			
Albumin level	33.840 ± 2.414 (*n* = 12)	36.010 ± 0.615 (*n* = 10)	0.885
TG	2.498 ± 0.189 (*n* = 12)	2.373 ± 0.369 (*n* = 10)	0.025
TC	6.572 ± 0.307 (*n* = 12)	5.996 ± 0.401 (*n* = 10)	0.260
HDL	2.012 ± 0.085 (*n* = 12)	1.768 ± 0.119 (*n* = 10)	0.103
LDL	3.323 ± 0.240 (*n* = 12)	3.036 ± 0.354 (*n* = 10)	0.499
VLDL	1.019 ± 0.064 (*n* = 12)	0.966 ± 0.148 (*n* = 10)	0.730
Inflammation levels			
CRP	4.725 ± 0.831 (*n* = 12)	1.92 ± 0.625 (*n* = 10)	0.025

*Fasting blood glucose (mmol/L)/OGTT-2 h (mmol/L)/albumin level (g/L)/triglyceride level (mmol/L)/C-reactive protein level (mg/L)* Kolmogorov‒Smirnov test, *OGTT-1 h (mmol/L)/total cholesterol (mmol/L)/high-density lipoprotein (mmol/L)/low-density lipoprotein (mmol/L)/very low-density lipoprotein (mmol/L)* Unpaired t test, *GD* Gestational diabetes, *OGTT* Oral glucose tolerance test, *TG* Triglyceride, *TC* Total cholesterol, *HDL* High-density lipoprotein, *LDL* Low-density lipoprotein, *VLDL* Very low-density lipoprotein, *CRP* C-reactive protein

No significant differences in gestational age were observed between the GD patient group and the normal pregnant woman group. However, the gestational body mass index (BMI) of GD patients was greater than that of healthy pregnant women (Table 3).

**Table 3:** Gestational age and gestational BMI of GD patients and healthy pregnant women (BMI and weeks, mean ± SEM)

	GD patients	Normal pregnant women	*P* value
Gestational weeks	27.42 ± 0.557 (*n* = 12)	26.30 ± 0.518 (*n* = 10)	0.1635
Gestational BMI	23.02 ± 0.667 (*n* = 12)	20.820 ± 0.475 (*n* = 8)	0.0177

*BMI* Body mass index, *GD* Gestational diabetes

### Differences in microbial diversity among nonpregnant women of reproductive age, healthy pregnant women, and GD patients

**Fig. 2:**
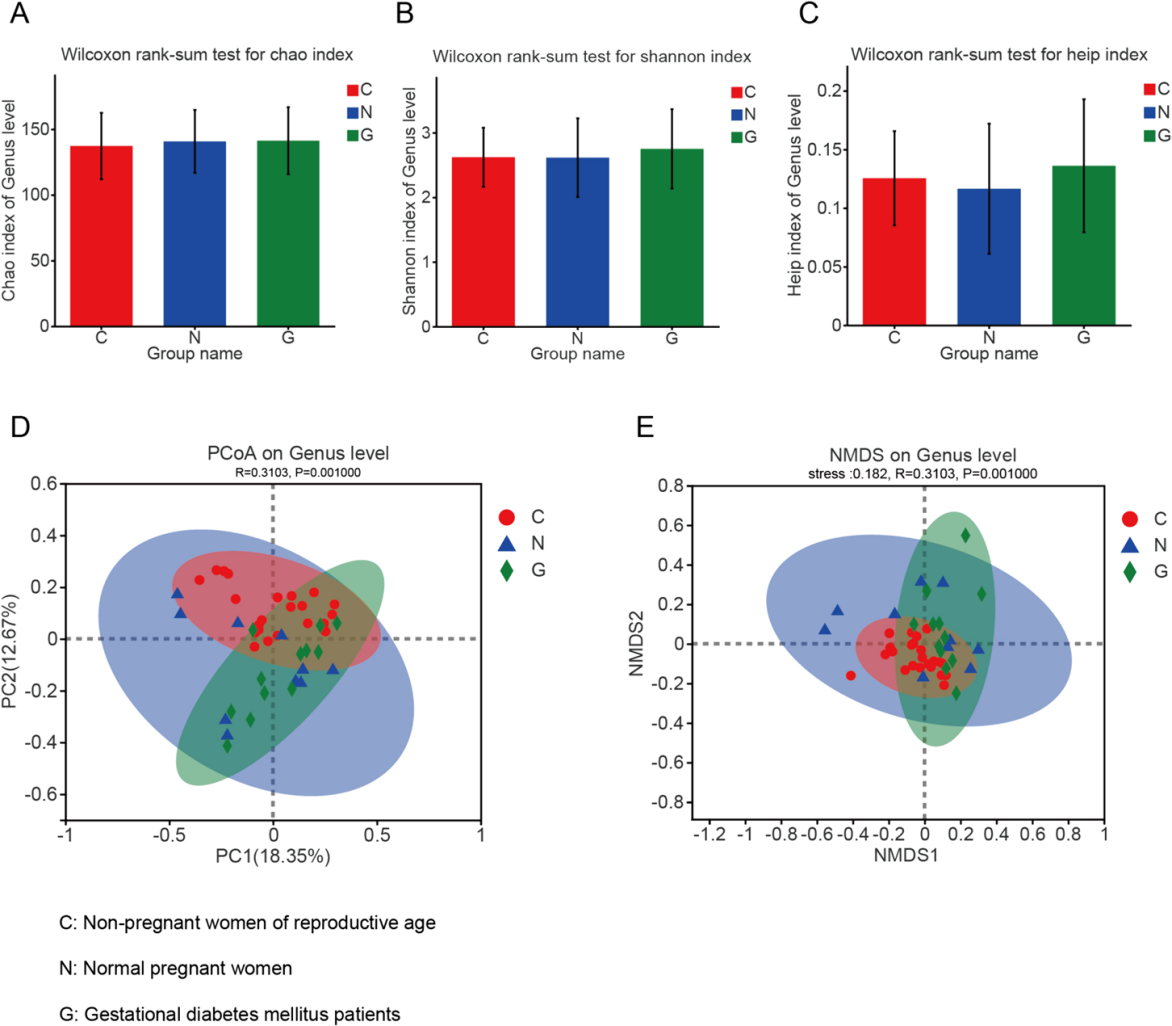
Diversity analysis of the intestinal microbiota in nonpregnant women of reproductive age, healthy pregnant women, and GD patients. The α-diversity analysis was based on the Chaos/Shannon/Heip index at the genus level (**A**, **B**, and **C**). β-Diversity analysis based on NMDS (**D**) and PCoA (**E**) at the genus level (**D** and **E**). Groups: C, nonpregnant women of reproductive age; N, normal pregnant women; G, gestational diabetes patients

Among the three groups, no significant differences were observed in intestinal microbiota richness (Chao1 index), diversity (Shannon index), or evenness (the Heip index) at the species, genus or phylum levels (Fig. 2A–C). Beta (β)-diversity analysis by PCoA and NMDS revealed significant differences in the overall composition of the gut microbiota among the three groups at the genus level (PCoA: R = 0.3103, *p* = 0.001; NMDS: R = 0.3103, *p* = 0.001) (Fig. 2D and E). However, differences in β diversity existed mainly between nonpregnant women of reproductive age and healthy pregnant women (PCoA: R = 0.4734, *p* = 0.001; NMDS: R = 0.001). 4734, *p* = 0.001), as well as between nonpregnant women of reproductive age and GD patients (PCoA: R = 0.2671, *p* = 0.001; NMDS: R = 0.2671, *p* = 0.001). No significant differences in β diversity were detected between healthy pregnant women and GD patients.

### Differences in microbial composition among nonpregnant women of reproductive age, healthy pregnant women, and GD patients

**Fig. 3:**
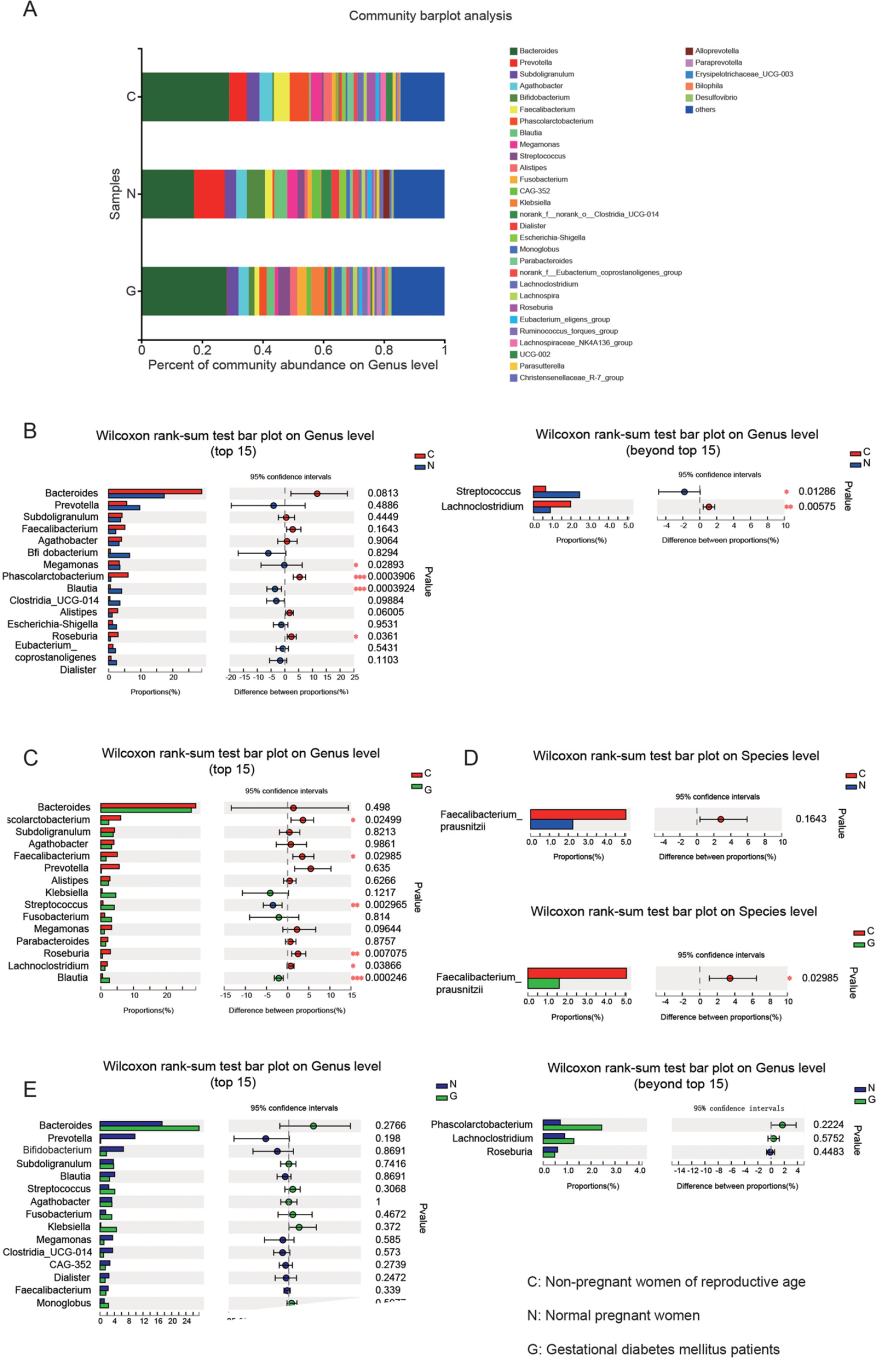
Differences in microbial composition at the genus level. **A** Genus distribution of the three groups presented by mean relative abundance (bar plot analysis). **B**–**E** Comparison of the relative abundance of the top 15 microbiota among the three groups at the genus level. Groups: C, nonpregnant women of reproductive age; N, normal pregnant women; G, gestational diabetes patients. * *p* < 0.05; ** *p* < 0.01; *** *p* < 0.001

A comparison of the community abundances of the 15 most common gut bacteria at the genus level among the three groups revealed that, between nonpregnant women of reproductive age and healthy pregnant women, significant differences in *Blautia*, *Phascolarctobacterium*, and *Roseburia* were observed (Fig. 3A-B). Compared to nonpregnant women of reproductive age, healthy pregnant women had an increase in the abundance of *Blautia* and a decrease in the abundances of *Phascolarctobacterium* and *Roseburia*. Additionally, comparisons between nonpregnant women of reproductive age and GD patients revealed some differences. In addition to the aforementioned bacteria, *Streptococcus*, *Faecalibacterium,* and *Lachnoclostridium* abundances were significantly different between these two groups. A marked increase in *Streptococcus* abundance and a decrease in *Faecalibacterium* and *Lachnoclostridium* abundances were observed in GD patients. (Fig. 3A and C). Further analysis revealed significant differences in *Streptococcus* and *Lachnoclostridium* abundances between nonpregnant women of reproductive age and healthy pregnant women, although these genera were not in the top 15 genera. Hence, there were differences in *Faecalibacterium* between healthy pregnant women and GD patients. However, the abundance of *Faecalibacterium* was not statistically decreased in healthy pregnant women compared to nonpregnant women, as it was in GD patients compared to nonpregnant women. At the species level, this *Faecalibacterium* was *Faecalibacterium prausnitzii* (Fig. 3D). A comparison of the community abundance of the 15 most common gut bacteria at the species level among the three groups was also performed (Extended Data Fig. 1).

Interestingly, the community abundance of the 15 most common gut bacteria, including *Faecalibacterium*, did not significantly differ between healthy pregnant women and GD patients (Fig. 3E).

Furthermore, we observed that gestation was associated with an increase in *Blautia* and *Streptococcus*. The increase in *Blautia* in GD patients was less than that in healthy pregnant women, and the increase in *Streptococcus* in GD patients was greater than that in healthy pregnant women. In addition, gestation decreased the abundance of *Phascolarctobacterium*, *Roseburia*, *Faecalibacterium*, and *Lachnoclostridium*. Herein, we observed that the decrease in *Roseburia* and *Faecalibacterium* in GD patients was greater than that in healthy pregnant women, and the decrease in *Phascolarctobacterium* and *Lachnoclostridium* in GD patients was less than that in healthy pregnant women.

**Fig. 4:**
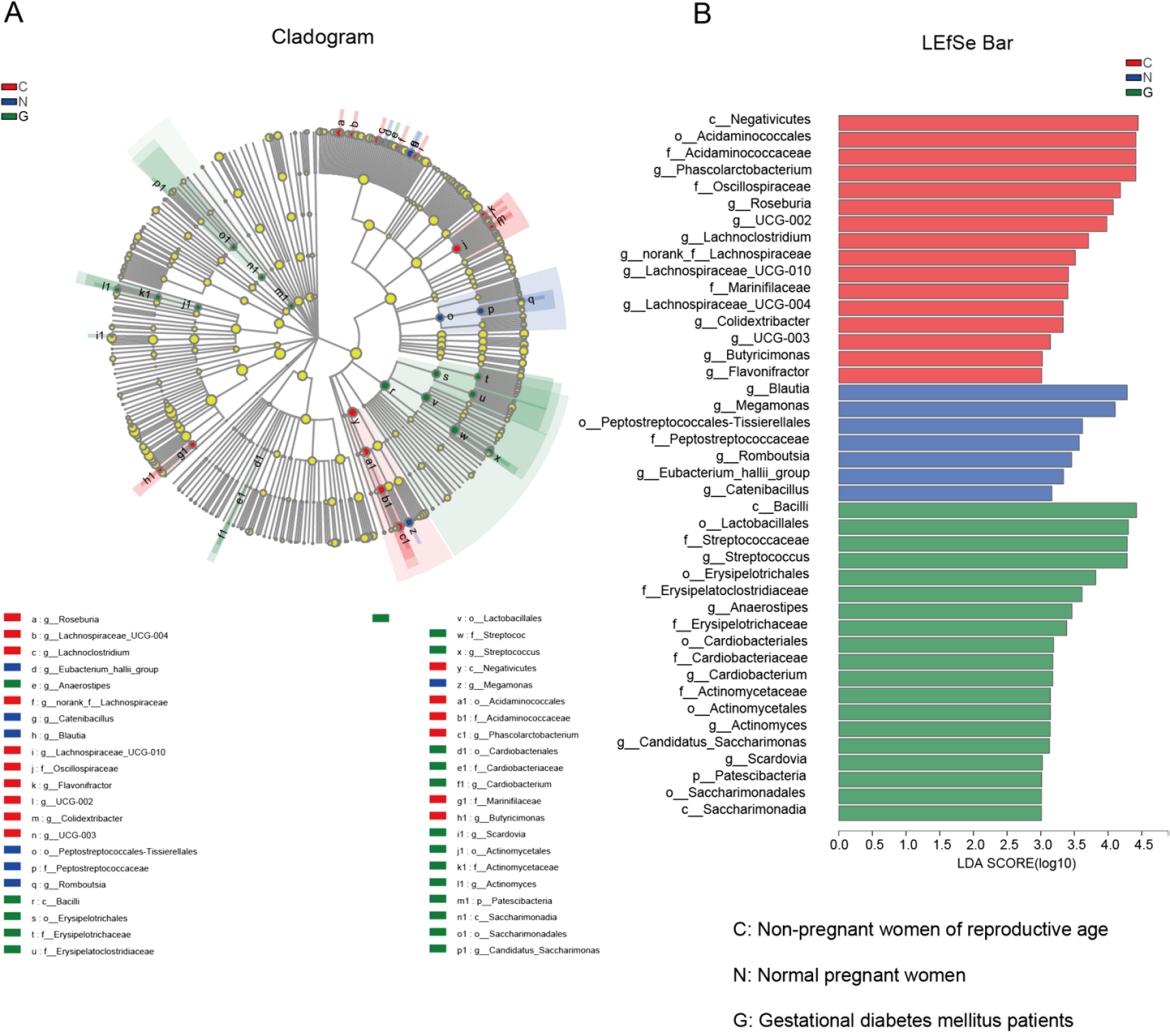
Differences in predominant gut bacteria at the genus level. **A** Cladogram of the predominant gut bacteria. **B** Differences in predominant gut bacteria analysed by LEfSe (LDA = 3). Red indicates gut bacteria enriched in nonpregnant women of reproductive age, blue indicates gut bacteria enriched in healthy pregnant women, and green indicates gut bacteria enriched in GD patients. Groups: C, nonpregnant women of reproductive age; N, normal pregnant women; G, gestational diabetes patients

The relative abundances of the predominant gut bacteria at the phylum to genus levels in the three groups were determined via a cladogram (Fig. 4A) and analysed via LEfSe (Fig. 4B). A significant difference in *Negativicute* among the three groups was detected. *Negativicute* was significantly enriched in the nonpregnant women group compared to the other groups. Moreover, the LDA score of *Negativicute* was greater than that of the other taxa in the nonpregnant women group, indicating that it was the most obvious biomarker differentiating it from the other groups. Similarly, *Blautia* and *Bacilli* were the most robust biomarkers for healthy pregnant women and GD patients, respectively.

### Correlation between glucose levels and altered gut bacteria in GD patients

**Fig. 5:**
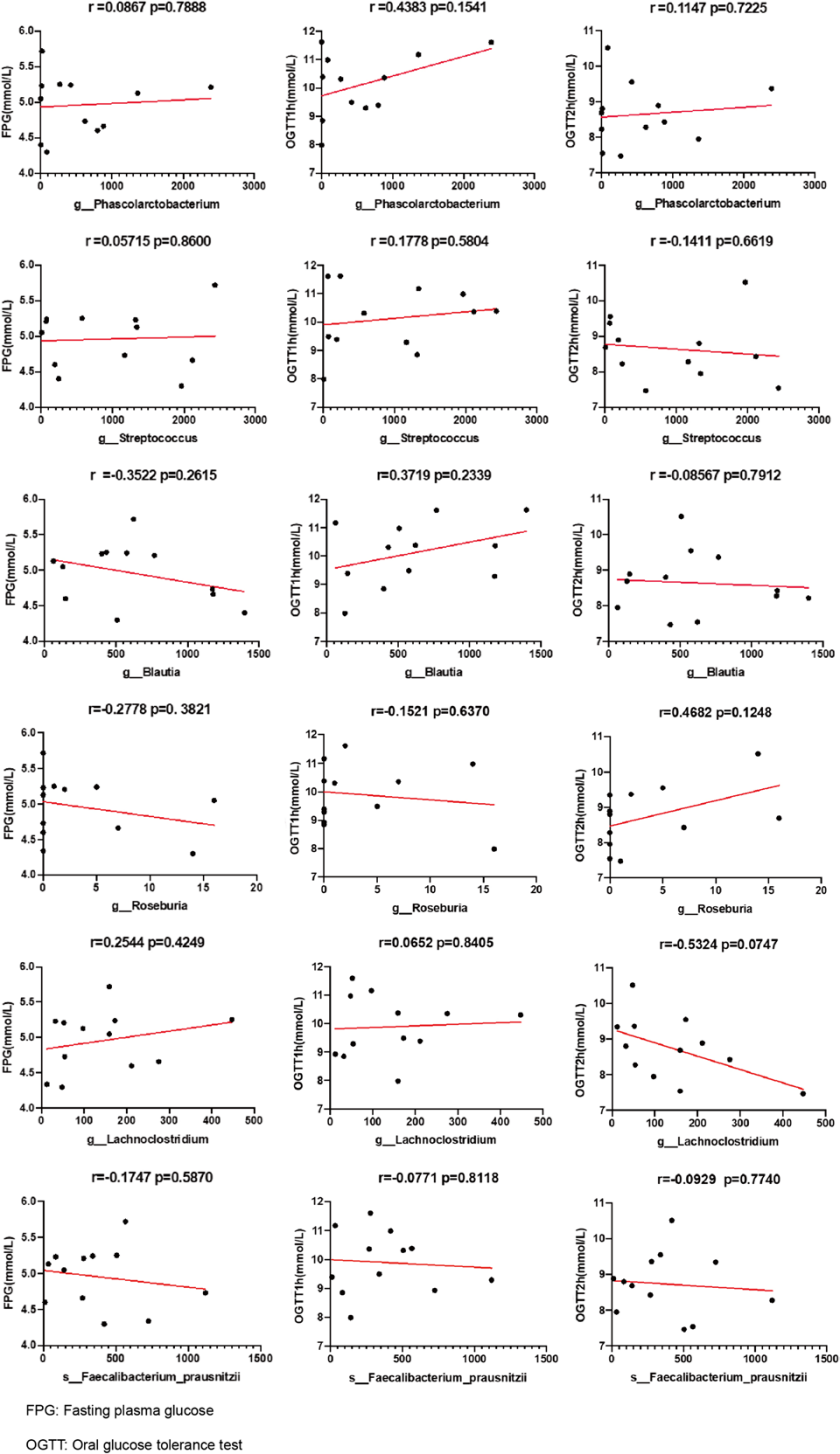
Correlations between the discrepant genera and fasting plasma glucose levels, plasma glucose levels after the OGTT 1 h, and plasma glucose levels after the OGTT 2 h

We also analysed the correlation between glucose levels and the relative abundances of discrepant genera via Pearson’s correlation analysis (Fig. 5). No significant correlation was detected between discrepant genera and glucose levels.

## Discussion

This was a cross-sectional study that demonstrated the coexistence of GD and an abnormal abundance of specific intestinal microbiota. The possibility that pregnancy itself could alter the microbiota has been revealed in previous studies. As the trimester progresses, there is a noticeable increase in microbial diversity, highlighting gradual alterations in diversity. For example, the relative abundance of *Bacteroidetes* decreased, while that of *Proteobacteria* increased [14]. Because of the increase in demand for energy storage, there is also an increase in the abundance of *Akkermansia*, *Bifidobacterium*, and *Firmicutes*. In addition, the increase in the relative abundance of *Proteobacteria* and *Actinobacteria* can serve as a protective factor against proinflammatory mechanisms [15]. Considering the inherent aspects of pregnancy, although there has been research on the differences in microbiota between healthy pregnant women and GD patients, the discrepancies in microbiota between healthy nonpregnant women (child-bearing age) and these two pregnancy statuses are equally important. However, these differences have yet to be explored. This study is the first to demonstrate the different compositions of the microbiota in these three population groups.

### Discoveries from the analysis of characteristics

First, we analysed the *personal information* of the patients in these groups and found that age, educational background, and family history of diabetes or hypertension were similar, and the gestational BMI differed between the two pregnancy groups. The GD patients had a high pregestational BMI. This result was consistent with previous findings showing that women with a high BMI had a markedly high risk of developing GD [16, 17]. Further analysis of clinical parameters revealed that the GD group had abnormal OGTT results rather than fasting blood glucose levels. This phenomenon seemingly indicated that postprandial insulin secretion rather than basal insulin secretion was disrupted in a majority of GD patients. However, our research has not yet explored this phenomenon further, and this result might be attributed to the small sample size. Gestation can induce a high pancreatic load because of metabolic adaptation to the nutritional requirements of the placenta and foetus. Hormones and growth factors derived from the placenta are key factors involved in altered pancreatic morphology and function [10]. This might stimulate the occurrence of disease in the subhealthy population. Hence, we speculated that people with impaired glucose tolerance before pregnancy have a greater risk of developing GD after pregnancy than do those with impaired fasting glucose before pregnancy. Thus, individuals with impaired glucose tolerance should receive stringent health management before pregnancy and strict blood sugar monitoring after pregnancy. According to these analyses, GD patients had a high level of TG, rather than TC or CRP. We also hypothesized that hypertriglyceridaemia was a prior risk factor for hypercholesteremia and could be a sensitive indicator during health screening. Moreover, a correlation between GD and inflammation has already been reported, and our results are consistent with previous findings [18, 19].

### Discoveries from the analysis of microbiology

Regarding the microbiological performance of the gut microbiota, previous studies have revealed that the dynamics of the gut microbiota differ from early to midgestation in GD patients but not in normoglycaemic pregnant women. For example, the phylum *Firmicutes* obviously decreases in abundance, which results in abnormal glucose tolerance [20, 21]. This phenomenon was also detected in our research, although the differences were not significant.

Although the correlation between the gut microbiota and GD was established several years ago, few studies have focused on the differences in the gut microbiota between nonpregnant women and pregnant women of these two pregnancy statuses. The current study investigated these three populations and revealed differences in the abundance of microbiota constituents induced by abnormal pregnancy, considering the influence of pregnancy itself at the same time.

### α-Diversity and β-Diversity

α-Diversity refers to the average species diversity within a region of the environment. Our research showed that there were no significant differences in intestinal microbiota richness, diversity or evenness among the three groups at the species, genus or phylum levels. However, differences in β-Diversity were identified. β-Diversity reflects the similarity of microbial composition between different samples. This study revealed significant differences in the composition of the gut microbiota between nonpregnant patients and pregnant patients (normal or abnormal). Unfortunately, no significant difference was observed between the normal pregnancy and GD pregnancy statuses. This might imply that pregnancy itself could induce obvious remodelling of the microbiota and that GD itself was unlikely to have a significant effect. Moreover, the effect of pregnancy may mainly exist on the component ratio rather than richness, diversity or evenness. However, it is important to note that the small sample size could also be a contributing factor.

### Faecalibacterium prausnitzii

Compared with nonpregnant women, GD patients showed a significant decrease in the abundance of *Faecalibacterium prausnitzii*; however, similar results were not observed in women with a healthy pregnancy. *Faecalibacterium prausnitzii* can produce microbial anti‐inflammatory molecules (MAMs) [22]. A recent study revealed the impaired structure and function of the intestinal barrier in diabetic mice. *Faecalibacterium prausnitzii* can strengthen the tight junctions of the colonic epithelium and repair the intestinal barrier [23]. Thus, the low abundance of *Faecalibacterium prausnitzii* in GD patients might indicate a bacterial impairment attributed to GD, leading to a weakened gut barrier and increased susceptibility.

### Blautia

The probiotic functions of *Blautia* have been characterized in the mammalian intestine [24]. Our study revealed an increase in the abundance of *Blautia* in healthy pregnant women. However, this variation was limited by GD status, although this difference was not statistically significant. A study proposed that depletion of *Blautia* species, especially *Blautia luti* and *Blautia wexlerae* species, contributes to metabolic inflammation and subsequent insulin resistance [25]. Therefore, we speculated that an increase in *Blautia* abundance during pregnancy was a protective factor, and a low abundance of a specific species of *Blautia* could be a biomarker for identifying individuals at high risk of GD.

### Streptococcus

Pregnancy also increased the abundance of *Streptococcus*. The current findings indicated an increase in the abundance of these bacteria in GD patients. Heat-killed *Streptococcus thermophilus* reinforces the immunity of the intestinal mucosa, inhibits inflammation, and improves glycaemic parameters in diabetic rats [26]. Conversely, intraperitoneal injection of *Streptococcus dysgalactiae subsp. Equisimilis* drastically increased blood glucose levels in C57BL6/J mice and reduced the survival rate of diabetic mice [27]. Hence, different species of *Streptococcus* could produce different effects on blood glucose levels. Nonetheless, our study could not identify the altered species of *Streptococcus*.

### Roseburia

*Roseburia* abundance decreased significantly during pregnancy, and the decrease in abundance in GD patients was greater than that in healthy pregnant women, indicating a severe deficiency of *Roseburia* in GD patients. *Roseburia* is a butyrate-producing genus. In minor stroke patients, it is negatively correlated with fasting glucose levels [28]. Another study demonstrated that the genus *Roseburia* might influence glucose metabolism in pregnant women and increase serum ketone levels [29]. Several studies on diabetes have shown a decreasing trend in the abundance of *Roseburia* type 1 or type 2 [30–33]. These phenomena might be attributed to the role of butyrate in glucose homeostasis [34]. Thus, the excessive reduction in *Roseburia* abundance in GD patients was also a result of bacterial impairment, which might be a key factor leading to disrupted glucose metabolism. Thus, replenishing *Roseburia* could be a viable approach to prevent GD.

### Phascolarctobacterium and lachnoclostridium

Gestation decreased the abundance of *Phascolarctobacterium* and *Lachnoclostridium*. Our study showed that the shrinkage of these two microbes in GD patients was less than that in normal late-pregnant women. *Phascolarctobacterium* species are succinate-utilizing bacteria that produce short-chain fatty acids and are closely related to the metabolic state [35]. *Lachnoclostridium* is a cutC-containing genus that can transform choline to trimethylamine and promote the development of atherosclerosis [36]. However, whether it is directly related to blood glucose has yet to be determined.

### Limitations and strengths

This study has several limitations. We were unable to identify whether the specific gut microbiota species that induce GD or that the GD status disturbed the composition of the gut microbiota. The gestational body mass index (BMI) of GD patients was greater than that of healthy pregnant women in our study. According to previous studies, the intestinal microbial composition of the obese population is significantly different from that of the normal population, and these differences are mainly manifested as a decrease in *Bacteroidetes* abundance and an increase in *Firmicutes* abundance [37, 38]. This finding suggests that obesity alone can lead to an imbalance in the microbiota. In contrast, we observed an increase in *Bacteroidetes* abundance and a decrease in *Firmicutes* abundance in GD patients. We are more inclined to interpret the disturbance in the intestinal microbiota as a result of GD rather than a result of obesity. Thorough studies are necessary in the future. Additionally, the sample size was limited. The main strengths of this study are that we added a group of nonpregnant women of reproductive age to elucidate which altered gut microbiota abundances resulted from normal pregnancy. Based on these findings, when we observed certain deficient gut microbiota in GD patients, we could distinguish whether the deficiency was a result of a lack of protective compensation or appearance after damage. The gut microbiota belonging to the former could be a potential genus that may regulate blood glucose in pregnant women. In future studies, we aim to explore the functions of these microbes by gavage or microbial transplantation in pregnant mouse models. Additionally, the optimal dosage and dosage form should be estimated for clinical applications.

## Conclusions

An abnormal increase or decrease in the gut microbiota, especially *Faecalibacterium prausnitzii*, was observed in GD patients.

### Supplementary Information

**Supplementary Material 1.**

## Data Availability

Database: science data bank. Accession numbers: 3191030@zju.edu.cn. Password: Flj123456. https://doi.org/10.57760/sciencedb.07951

## Abbreviations

GD: Gestational diabetes
OGTT: Oral glucose tolerance test
PCR: Polymerase chain reaction
OUT: Operational taxonomic unit
SEM: Standard error of the mean
PCoA: Principal coordinates analysis
NMDS: Non-metric multidimensional scaling
LEfSe: Linear discriminant analysis effect size
BMI: Body mass index
TG: Triglyceride
TC: Total cholesterol
HDL: High density lipoprotein
LDL: Low density lipoprotein
VLDL: Very low density lipoprotein
CRP: C-reactive protein
