# Supplementary figures and images for: Imbalance of gut microbiota in gestational diabetes
Source: BMC Pregnancy Childbirth. 2024 Apr 1;24:226. doi: 10.1186/s12884-024-06423-0 (PMC10983739; doi:10.1186/s12884-024-06423-0)

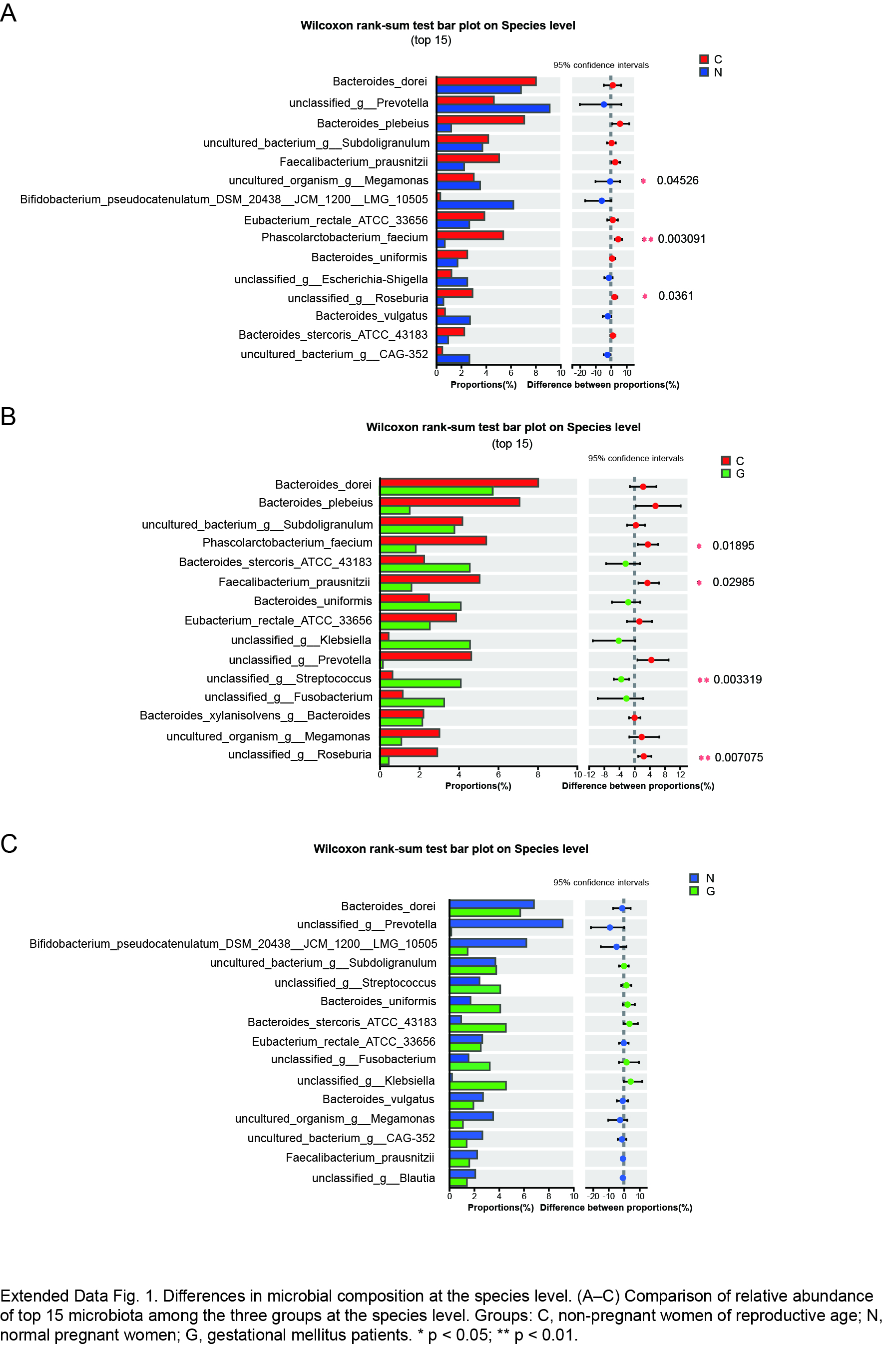

Supplement: Supplementary file 1 — Supplementary Material 1. [file 12884_2024_6423_MOESM1_ESM.jpg]
